# Urban–rural differences in BMI status, 24-hour movement guideline compliance, executive functions, and motor skills in preschool children: a cross-sectional study

**DOI:** 10.3389/fphys.2026.1882530

**Published:** 2026-08-05

**Authors:** Mohamed Amine Ltifi, Sonia Meksi, Abdulazeem Alotaibi, Mohamed Souhaiel Chelly

**Affiliations:** 1 University of Gafsa, Higher Institute of Sport and Physical Education of Gafsa, Gafsa, Tunisia; 2 Research Laboratory (LR23JS01) « Sport Performance, Health & Society», Higher Institute of Sport and Physical Education of Ksar Said, University of Manouba, Tunis, Tunisia; 3 Department of Physical Education and Kinesiology, College of Education, Qassim University, Qassim, Saudi Arabia

**Keywords:** BMI, executive functions, motor skills, physical activity, preschool children, sedentary behavior, urban–rural differences

## Abstract

**Background:**

Early childhood is a critical developmental period during which weight status, 24-hour movement behaviors (MB), executive functions (EF), and motor skills (MS) interact to influence long-term health and development. However, few studies have simultaneously examined these domains among preschool children in North African countries, particularly considering urban–rural differences. Therefore, this study investigated urban–rural differences in body mass index (BMI) categories, 24-hour MB, EF, and MS among Tunisian preschool children.

**Methods:**

A cross-sectional observational study was conducted in Tunisian preschool children. The sample included 112 children, of whom 74 were from urban areas and 38 from rural areas (50 boys and 62 girls), aged 4–5 years (mean age: 4.10 ± 0.58 years). BMI categories were defined according to World Health Organization (WHO) standards. Anthropometric measurements were used to calculate BMI-for-age z-scores and classify children as below normal, normal, or above normal. Twenty-four-hour MB, including physical activity (PA), sedentary behavior (SB), and sleep, were objectively assessed using accelerometers over five consecutive days. EF (inhibition and working memory) were assessed using standardized cognitive tests. Gross MS were evaluated using the Supine Timed Up and Go, One-Leg Standing Balance, Hand Grip Dynamometer, and Standing Long Jump tests, while fine MS were assessed using the 9-Hole Pegboard Test.

**Results:**

Significant differences between BMI categories were found for most anthropometric variables. Children with BMI above normal showed higher body weight and BMI-for-age z-scores. No significant differences were observed for accelerometer-derived MB, EF, or MS. However, BMI categories were associated with adherence to some 24-hour movement recommendations. While 76.4% met physical activity guidelines and 81.3% met sleep recommendations, only 18% adhered to all integrated guidelines.

**Conclusions:**

These findings suggest associations between BMI categories and anthropometric differences, while no clear associations were observed with variations in MB, EF, or motor competence. Urban–rural differences in adherence to some movement recommendations suggest a potential role of environmental context in children’s daily behaviors. Given the cross-sectional and observational design, results should be interpreted with caution. Larger longitudinal studies are needed to clarify the complex interactions between weight status, MB, cognitive development, and MS in early childhood.

## 1 Introduction

Early childhood (3–6 years) represents a critical period for motor, cognitive, and behavioural development, with significant implications for future health and lifestyle habits (Best and Miller, 2010; Diamond, 2013). During this phase, children rapidly acquire fundamental capacities such as basic motor skills (MS), self-regulation, and early cognitive processes, which form the foundation for school readiness and long-term physical health (National Research C and Institute of Medicine Committee on Integrating the Science of Early Childhood D, 2000; Alonso-Martínez et al., 2025).

Twenty-four-hour movement behaviours (MB), encompassing physical activity (PA), sedentary time, and sleep, play a central role in this developmental period (Barros et al., 2021; Fernandes et al., 2022). Regular PA is associated with enhanced MS and overall functional development, whereas excessive sedentary behaviour (SB) is linked to an increased risk of overweight and reduced motor performance (Krupsky et al., 2020; Whiting et al., 2021; Ltifi et al., 2026). Sleep and nap practices also contribute to neurocognitive development and body weight regulation (Chaput et al., 2014; Kuzik and Carson, 2016; Draper et al., 2020; Ltifi et al., 2025b). Recent research highlights the importance of considering these behaviours as an integrated framework rather than isolated components, as their quality and distribution throughout the day can influence neuro-motor outcomes (Martí-Nicolovius, 2022; Lau et al., 2024).

Body weight status, often assessed by body mass index (BMI) according to World Health Organization (WHO), is a major determinant of motor and cognitive development (Group, 2006a; Group, 2006b; de Onis et al., 2007; WHO Guidelines Approved by the Guidelines Review Committee, 2019; Okely et al., 2021). Deviations from normal BMI, including underweight or overweight, can affect motor competence, participation in PA, and executive function performance (Greier and Drenowatz, 2018; Lima et al., 2021; Rico-González et al., 2024). Motor competence has been identified as a key mediator between weight status and engagement in PA, with biomechanical and physiological constraints potentially making certain movements more challenging, thereby influencing motor learning and self-confidence (Barnett et al., 2016; Fernandes et al., 2022).

Beyond motor aspects, weight status also appears to be linked to cognitive development, particularly executive functions (EF) such as inhibition, working memory, and cognitive flexibility (Best and Miller, 2010; Diamond, 2013; Blair and Raver, 2015; Ltifi et al., 2025a; Ltifi et al., 2025c; Ltifi et al., 2026). Preschool children with excess weight often exhibit lower EF and motor performance compared with their normal-weight peers (Li et al., 2008; Mamrot and Hanć, 2019). Moreover, early environmental factors, including home and school stimulation, parental education, and socioeconomic status, can modulate the impact of weight status and daily behaviours on cognitive and motor outcomes (de Souza Morais et al., 2021; Ltifi et al., 2025c).

Despite these insights, few studies have simultaneously examined BMI, 24-hour MB, motor competence, and EF within a comprehensive analytical framework, particularly in low- and middle-income countries such as Tunisia (Ltifi et al., 2025c; Ltifi et al., 2025d; Ltifi et al., 2026). Existing evidence remains somewhat inconsistent, with some studies reporting associations between excess weight and reduced motor or cognitive function, while others report no significant relationships (Barros et al., 2021; Alonso-Martínez et al., 2025; Pacheco et al., 2025). These inconsistencies underscore the need for multidimensional research approaches that integrate weight status, daily movement patterns, motor competence, cognitive development, and environmental influences.

Residential context is increasingly recognized as an important ecological determinant of child development. According to ecological models of health and development, children’s movement behaviours, motor competence, and EF are shaped not only by individual characteristics but also by the physical and social environments in which they live (Draper et al., 2020; Okely et al., 2021). Urban and rural settings may differ substantially in terms of access to safe outdoor play spaces, availability of recreational facilities, opportunities for structured and unstructured physical activity, transportation patterns, parental supervision, socioeconomic conditions, and early childhood education resources (Ltifi et al., 2025c). These environmental characteristics can influence children’s daily movement behaviours, opportunities for motor skill practice, and cognitive stimulation, thereby potentially modifying the associations between BMI, movement behaviours, motor competence, and EF (Okely et al., 2021). Such contextual influences may be particularly relevant in low- and middle-income countries, where disparities in infrastructure and access to health and educational resources between urban and rural communities are often more pronounced.

Of particular interest are potential differences between children living in urban versus rural settings. Recent Tunisian research, including the SUNRISE study, suggests that daily routines, access to play spaces and structured activities, as well as cultural and educational practices, can significantly influence MB, MS, and EF (Okely et al., 2021; Ltifi et al., 2025c; Meksi et al., 2026). Understanding urban–rural variations is essential for designing targeted interventions that address the specific needs of children according to their living environment.

In this context, the present cross-sectional study aimed to investigate urban–rural differences in BMI categories, compliance with 24-hour movement guidelines, executive functions (EF), and motor skills (MS) among Tunisian preschool children. We hypothesized that BMI would be associated with movement behaviours, motor competence, and EF and that these associations could vary according to residential setting (urban versus rural). Accordingly, analyses were stratified by residential setting to explore potential contextual differences. Because of the limited sample size within several BMI subgroups, these analyses were considered exploratory, and no formal statistical interaction analysis was performed. To our knowledge, no previous study has concurrently examined these four domains in Tunisian preschool children, highlighting the novel contribution of this research to understanding neuro-motor development across different residential contexts.

## 2 Materials and methods

### 2.1 Participants

This observational cross-sectional study included 112 preschool children, 74 children were from urban areas and 38 from rural areas, (50 boys and 62 girls) aged 4 to 5 years (mean age: 4.10 ± 0.58 years; median: 4.13 years). Participants were recruited from five kindergartens located in urban and rural areas of the Greater Tunis region, Tunisia: El Manar and El Omrane (urban), and Cité Zahrouni and Séjoumi (rural).

Of the 112 children, 74 were from urban areas and 38 from rural areas, reflecting the study’s primary variable of interest, residential setting. This distribution was considered in order to examine potential urban–rural differences in anthropometry, MB, MS, and EF.

The selected kindergartens enrolled children aged 4–5 years with parental or legal guardian consent. Children with medical conditions, physical disabilities, developmental disorders, or any condition potentially affecting movement, MS, or EF were excluded. The selection of kindergartens was based on feasibility and willingness to participate, while ensuring representation from both urban and rural areas of the Greater Tunis region. Kindergartens were chosen to capture variability in environmental and sociodemographic contexts. Participants were recruited through a school-based recruitment process. All children enrolled in the selected kindergartens and meeting the age criteria (4–5 years) were invited to participate. Information sheets and consent forms were distributed to parents or legal guardians through the kindergarten administration. Only children whose parents provided written informed consent were included in the study.

Although urban–rural differences are well documented, data from both settings were combined to increase statistical power and provide a sample broadly representative of preschool children in the Greater Tunis region, while minimizing potential confounding.

Kindergartens were randomly selected within urban and rural areas to ensure a diverse sample. All eligible children within participating kindergartens were invited to participate. Twenty-eight children and their parents declined participation, resulting in an overall participation rate of approximately 81%. While the sample is not fully nationally representative, it reflects typical sociodemographic characteristics of preschool children in these regions.

It should be noted that preschool attendance in Tunisia is not universal, and children enrolled in kindergartens may differ from non-enrolled peers, particularly in terms of socioeconomic status, parental education, and access to early educational resources. Therefore, the present sample may be slightly biased toward families who have access to and choose formal preschool education. This limits the generalizability of the findings to all Tunisian preschool children. However, within the Greater Tunis region, kindergarten-based sampling remains a commonly used and practical approach for accessing this age group in a structured educational setting.

### 2.2 Procedure

All assessments were conducted within the kindergarten setting by a trained research teacher and two research assistants, following standardized data collection protocols. Anthropometric measurements, motor skill assessments, and executive function tasks were administered individually. Children wore accelerometers for five consecutive days during their daily activities. The order of assessments was standardized across all kindergartens.

On the first day, children were fitted with the accelerometer and anthropometric measurements were assessed. On the second day, gross motor skill tests were conducted in the following sequence: functional mobility (Supine Timed Up and Go), postural balance (one-leg standing), upper body strength (handgrip dynamometry), and lower body strength (standing long jump). On the third day, fine motor dexterity (9-Hole Pegboard Test) and the inhibition task (Mr. Ant) were administered. On the fourth day, the working memory task (Go/No-Go) was conducted, and accelerometers were removed at the end of the day (Friday).

After each assessment, children were provided with adequate rest while other participants completed their evaluations to minimize fatigue and potential learning effects. Parent and kindergarten questionnaires were administered via face-to-face interviews at times convenient for families and staff.

### 2.3 Anthropometry and BMI Classification

#### 2.3.1 Anthropometry

Body height was measured to the nearest 0.1 cm using customizable and repositionable adhesive measuring tapes, following standardized anthropometric procedures. Children stood barefoot in an upright position with the head aligned according to the Frankfurt plane. Each measurement was performed twice, and if the difference between the two readings exceeded 0.5 cm, a third measurement was taken. The mean value was retained for analysis.

Body mass was assessed to the nearest 0.1 kg using a calibrated SECA 750 Viva scale (SECA, Hamburg, Germany), with children wearing light clothing and no shoes. Two measurements were obtained, and if the difference exceeded 0.25 kg, a third measurement was conducted, and the average value was used. BMI was calculated as body weight (kg) divided by height squared (m²). Height-for-age (HAZ), weight-for-age (WAZ), and BMI-for-age (BAZ) z-scores were calculated based on WHO growth reference standards (Group, 2006b).

#### 2.3.2 BMI classification

According to WHO BMI-for-age reference standards (Lima et al., 2021; Alonso-Martínez et al., 2025), children were initially classified as follows: severe thinness (< −3 SD), thinness (−3 to < −2 SD), normal weight (−2 to +1 SD), at risk of overweight (> +1 to +2 SD), overweight (> +2 to +3 SD), or obesity (> +3 SD).

For statistical analyses, BMI categories were consolidated into three groups:

- BMI below normal (severe thinness and thinness)
- BMI normal
- BMI above normal (at risk of overweight, overweight, and obesity)

This classification is consistent with WHO standards for children aged 0–60 months (WHO Anthro) and 61 months to 19 years (WHO AnthroPlus) (de Onis et al., 2007; Okely et al., 2021), ensuring a standardized assessment of weight status adjusted for age and sex.

In the present sample, 74 children were from urban areas and 38 from rural areas. The distribution of children across BMI categories was as follows (Ltifi et al., 2025d):

Urban: BMI < normal: n = 2; BMI = normal: n = 38; BMI > normal: n = 34
Rural: BMI < normal: n = 3; BMI = normal: n = 31; BMI > normal: n = 4

This distribution reflects the main study variable, namely residential setting.

### 2.4 Physical activity, sedentary behaviour, and sleep

PA, SB, and sleep were objectively assessed using ActiGraph GT3X accelerometers (ActiGraph LLC, USA) worn on the right hip for five consecutive days. Accelerometers were initialized at a 30 Hz sampling rate, and data were reintegrated into 15-second epochs using a low-frequency filter. Data processing was conducted with ActiLife software (version 6.1.2.1). A valid day was defined as at least 24 hours of recorded data, including a minimum of 6 hours of valid waking wear time.

Non-wear periods were defined as 20 or more consecutive minutes of zero counts and were excluded from analyses. Sleep periods were identified from accelerometer data and corroborated with parental reports and activity logs; these periods were excluded from PA and SB analyses. Activity intensity thresholds were applied as follows: SB (<800 counts·min^-1^), light-intensity PA (800–1679 counts·min^-1^), moderate-intensity PA (1680–3367 counts·min^-1^), and vigorous-intensity PA (≥3368 counts·min^-1^) (Pate et al., 2006; Pate et al., 2015).

Prior to data collection, a familiarization session was conducted with all children and their parents/guardians to explain the use of the ActiGraph devices and ensure proper acceptance. No difficulties, discomfort, or behavioral disturbances related to wearing the accelerometers were reported during the monitoring period.

### 2.5 Executive function

Executive function was assessed using the Early Years Toolbox (Blair and Raver, 2015), focusing on behavioural impulse control and inhibition (Go/No-Go task), as well as visuospatial working memory (Mr. Ant task). Each task lasted approximately 10 minutes and included a standardized practice phase at the beginning.

The original French versions of the tasks were translated into Arabic using a forward-translation procedure by a bilingual fieldworker who is also a physical education teacher. Before formal testing, each child completed the standardized practice phase to ensure comprehension of the task instructions. During data collection, no child showed difficulties understanding the instructions or completing the tasks, and no administration problems were observed by the trained research assistants. The Arabic version is currently undergoing formal psychometric validation among Tunisian preschool children (Ltifi et al., 2025c; Ltifi et al., 2025d), however, previous studies in similar contexts have confirmed its feasibility, acceptability, and comprehensibility.

All EF assessments were conducted individually in a quiet setting within the kindergartens by trained research assistants following standardized administration procedures. Although the original Early Years Toolbox has demonstrated good reliability and validity in international preschool populations (Ltifi et al., 2025a; Ltifi et al., 2025c; Ltifi et al., 2025d), the ongoing validation of the Arabic version should be considered when interpreting the executive function findings, particularly the absence of significant associations (Ltifi et al., 2025a; Ltifi et al., 2025c; Ltifi et al., 2025d).

### 2.6 Gross and fine motor skills

MS were assessed using selected tests from the NIH Toolbox (Gershon et al., 2013) in a spacious classroom environment. These assessments have demonstrated strong reliability and validity in international studies (Leppänen et al., 2019; Delisle Nyström et al., 2020; Draper et al., 2020; Ltifi et al., 2025d) with preschool children, supporting their use for evaluating both gross and fine MS.

MS was analysed using the raw scores obtained for each assessment following the standardized NIH Toolbox administration procedures. No normative reference values derived from other populations were applied; therefore, the analyses were based exclusively on each child’s observed performance, minimizing potential bias related to cross-cultural normative comparisons.

#### 2.6.1 Gross motor skills

##### 2.6.1.1 Functional mobility: supine timed up and go

Functional mobility was evaluated using the supine Timed Up and Go test. Children began lying on their back behind a marked starting line, stood up as quickly as possible, ran to touch a target located 3 meters away, and returned to the starting position. One practice trial and two recorded test trials were performed, with the mean value used for analysis (Okely et al., 2021; Ltifi et al., 2025a).

##### 2.6.1.2 Postural stability: one-leg standing balance test

Postural control was assessed via the one-leg standing balance test. Children stood on one leg with arms relaxed alongside the body, while the opposite foot was lifted off the ground. The trial ended if the child shifted the standing foot or wrapped the free leg around it. Each leg was tested for a maximum of 30 seconds, and the mean of both legs was calculated for analysis.

##### 2.6.1.3 Upper body strength: hand grip dynamometry

Upper body strength was measured using a hand grip dynamometer (TKK 5825, Grip-A). Children were instructed to squeeze the device with maximum effort for at least 3 seconds without contacting the body. One practice trial and two recorded trials were performed for each hand, with the mean value retained for analysis (Mathiowetz et al., 1986).

##### 2.6.1.4 Lower body strength and mobility: standing long jump

Lower limb strength and functional mobility were assessed using the standing long jump. Participants stood behind a marked line and jumped forward as far as possible using both feet. After a practice jump, two trials were recorded, and the mean distance was used for analysis.

#### 2.6.2 Fine motor skills

##### 2.6.2.1 Manipulation: 9-hole pegboard test

Fine motor dexterity was evaluated using the 9-Hole Pegboard Test. Children were instructed to place and remove nine pegs sequentially as quickly as possible. Timing began when the first peg was touched and ended upon removal of the final peg. The total completion time (seconds) was used for analysis. This assessment has demonstrated high reliability and validity for evaluating fine MS in children (Smith et al., 2000).

### 2.7 Questionnaires

#### 2.7.1 Center information questionnaire

A Center Information Questionnaire was administered through interviews with kindergarten directors. This instrument captured information on the total number of enrolled children, participation rates, daily nap schedules, meal provision, food policies, and nutrition practices within each center.

Additionally, the questionnaire incorporated items adapted from the SUNRISE parent/guardian questionnaire to provide contextual data on children’s daily routines, sleep schedules, screen time, SB, and dietary intake outside the kindergarten setting. Caregivers were asked about their interactions with the child during meals, play, walks, travel, and bedtime routines.

The SUNRISE questionnaire was made available in both Arabic and French, allowing caregivers to select their preferred language. These data were used solely to provide descriptive contextual information about the kindergarten environment and were not included as covariates in the statistical analyses (Okely et al., 2021).

### 2.8 Statistical analysis

All statistical analyses were conducted using SPSS version 26.0 (IBM Corp., Armonk, NY, USA). Descriptive statistics were calculated for all variables, including means and standard deviations (SD) for continuous variables and frequencies and percentages for categorical variables. Normality of continuous data was evaluated using the Shapiro–Wilk test.

Given the relatively small sample sizes within some BMI subgroups and the non-normal distribution of several outcome variables, non-parametric tests were employed. Differences in anthropometric measures, 24-hour MB, executive function, and motor skill outcomes across BMI categories were tested using Kruskal–Wallis one-way analysis of variance. When significant overall differences were detected, pairwise comparisons were conducted with Bonferroni-adjusted p-values to account for multiple comparisons. To complement p-values and provide an estimate of the practical significance of the observed differences, epsilon squared (*ϵ*^2^) effect sizes were calculated for each analysis. Effect sizes were interpreted according to the following thresholds: negligible (<0.01), small (0.01–0.06), moderate (0.06–0.14), and large (>0.14) (Cohen, 1988).

Chi-square tests were used to examine differences in the proportion of children meeting the 24-hour movement guidelines across BMI categories within urban and rural settings. Fisher’s exact test was applied when expected cell counts were less than five.

All analyses were stratified by residential setting (urban vs. rural) to explore potential urban–rural differences. Statistical significance was set at p < 0.05. Given the observational and cross-sectional nature of the study, findings were interpreted as associational rather than causal.

No *a priori* sample size or power calculation was conducted for the present study, as the sample was determined by feasibility and availability of participants within participating kindergartens. Given the resulting small subgroup sizes, particularly within BMI categories stratified by residential setting, statistical power may be limited for detecting small-to-moderate effects.

Because of the limited sample size within several BMI subgroups, particularly in the rural cohort, formal interaction analyses between BMI category and residential setting were not performed, as these analyses would have been underpowered and potentially unstable. Therefore, urban–rural comparisons should be considered exploratory and hypothesis-generating rather than confirmatory.

## 3 Results

**Table 1** presents the anthropometric characteristics and 24-hour MB of Tunisian preschool children stratified by BMI categories within urban and rural settings.

**Table 1:** Anthropometric characteristics and movement behaviors stratified by BMI Categories by Region.

		Urban (74)					Rural (38)				
Variables	All (n = 112)	BMI < normal (n=2) [95% CI]	BMI = normal (n=38) [95% CI]	BMI > normal (n= 34) [95% CI]	P	ϵ²	BMI < nor mal (n= 3) [95% CI]	BMI = normal (n= 31) [95% CI]	BMI > normal (n= 4) [95% CI]	p	ϵ²
Age (years)	4,10 ± 0,58	3,81 ± 0,17	3,91 ± 0,49	3,88 ± 0,59	0.912	0.026	4,80 ± 0,29	4,54 ± 0,42	4,03 ± 0,53	0.080	0.888
Weight (kg)	18,60 ± 2,93	11,35 ± 1,77 [-4.55; 27.25]	17,87 ± 2,39 [17.08; 18.66]	20,36 ± 2,91 [19.34; 21.38]	0.356€ **0.022Π** **0.009β**	0.194	13,87 ± 1,10 [11.14; 16.60]	18,28 ± 1,95 [17.56; 19.00]	20,25 ± 3,07 [15.36; 25.14]	**0.022€** **0.009Π** 0.690β	0.212
Height (cm)	107,38 ± 6,76	96,50 ± 7,28 [31.09; 161.91]	106,65 ± 6,86 [104.40; 108.90]	105,12 ± 5,70 [103.13; 107.11]	0.164	0.023	107,03 ± 5,07 [94.44; 119.62]	111,33 ± 5,84 [109.19; 113.47]	108,55 ± 8,49 [95.04; 122.06]	0.352	0.002
BMI (kg/m²)	16,12 ± 2,00	12,15 ± 0,06 [11.61; 12.69]	15,65 ± 0,82 [15.38; 15.92]	18,36 ± 1,44 [17.86; 18.86]	1.000€ **0.002Π** **<0.001β**	0.761	12,10 ± 0,43 [11.03; 13.17]	14,74 ± 0,98 [14.38; 15.10]	17,12 ± 0,39 [16.50; 17.74]	**0.034€** **<0.001Π** **0.009β**	0.425
HAZ	0.85 ± 1.20	-2,53 ± 1,09 [-7.26; 12.32]	0,77 ± 0,91 [0.47; 1.07]	1,77 ± 0,87 [1.47; 2.07]	0.539€ **0.011Π** **<0.001β**	0.325	-1,87 ± 0,51 [0.60; 3.14]	0,35 ± 0,71 [0.09; 0.61]	1,55 ± 1,04 [-0.10; 3.20]	**0.026€** **0.001Π** 0.113β	0.297
WAZ	0.89 ± 1.26	-1,17 ± 1,46 [-11.95; 14.29]	1,05 ± 1,41 [0.59; 1.51]	0,76 ± 1,13 [0.37; 1.15]	0.139	0.027	-0,29 ± 0,84 [-1.80; 2.38]	1,05 ± 1,06 [0.66; 1.44]	1,23 ± 1,77 [-1.59; 4.05]	0.165	0.046
BAZ	0.48 ± 1.37	-2,71 ± 0,11 [1.72; 3.70]	0,22 ± 0,61 [0.02; 0.42]	1,96 ± 0,83 [1.67; 2.25]	1.000€ **0.002Π** **<0.001β**	0.761	-2,67 ± 0,42 [1.63; 3.71]	-0,42 ± 0,72 [0.16; 0.68]	1,28 ± 0,23 [0.91; 1.65]	**0.034€** **<0.001Π** **0.009β**	0.425
LPA (min/day) a	88.45 ± 24.75	–	83 ± 21,72 [75.86; 90.14]	89,24 ± 59,58 [68.45; 110.03]	-0.001	-0.015	82 ± 19,97 [32.39; 131.61]	91 ± 27,14 [81.04; 100.96]	115 ± 20,67 [82.11; 147.89]	0.139	0.056
MPA (min/day) a	61.74 ± 23.74	–	53 ± 20,39 [46.30; 59.70]	59,58 ± 23,24 [51.47; 67.69]	0.014	0.0	77 ± 18,03 [32.21; 121.79]	67 ± 24,70 [57.94; 76.06]	89 ± 14,84 [65.39; 112.61]	0.089	0.081
VPA (min/day) a	18.92 ± 12.81	–	17 ± 13,57 [12.54; 21.46]	16,66 ± 11,57 [12.62; 20.70]	-0.014	-0.028	17 ± 12,09 [-13.03; 47.03]	21 ± 12,63 [16.37; 25.63]	32 ± 10,60 [15.13; 48.87]	0.149	0.052
MVPA (min/day) a	80.66 ± 34.87	–	69 ± 32,80 [58.22; 79.78	76,24 ± 33,39 [64.59; 87.89]	0.003	-0.011	94 ± 27,89 [24.72; 163.28]	88 ± 35,47 [74.99; 101.01]	121 ± 21,61 [86.61; 155.39]	0.080	0.087
TPA (min/day) a	169.11 ±55.14	–	152 ± 51,99 [134.91; 169.09]	165.48 ± 50,29 [147.93; 183.03]	0.012	-0.002	176 ± 47,83 [57.18; 294.82]	178 ± 58,38 [156.59; 199.41]	235 ± 27,70 [190.92; 279.08]	0.072	0.093
SB (min/day) a	627.58 ± 55.75	–	645 ± 54,22 [627.18; 662.82]	620,86 ± 45,59 [604.95; 636.77]	0.039	0.025	631 ± 53,18 [498.89; 763.11]	624 ± 59,50 [602.18; 645.82]	567 ± 56,93 [476.41; 657.59]	0.166	0.045
SST (min/day)	70.54 ± 32.93	60,00 ± 0,00	69,24 ± 34,38 [60.00; 60.00]	74,74± 36,22 [57.94; 80.54]	0.706	-0.018	80,33 ± 38,11 [-14.34; 175.00]	70,23 ± 29,69 [59.34; 81.12]	47,50 ± 15,00 [23.63; 71.37]	0.242	0.024
Sleep (min/day)	638.79 ± 53.45	592,50 ± 31,82	650,37 ± 56,77 [306.61; 878.39]	636,38 ± 55,77 [631.71; 669.03]	0.122	0.031	610,33 ± 17,04 [568.00; 652.66]	633,61 ± 50,70 [615.01; 652.21]	633,75 ± 39,45 [570.98; 696.52]	0.702	-0.037

BAZ, BMI-for-age z-score; BMI, body mass index; HAZ, height-for-age z-score; LPA, light-intensity physical activity; MPA, moderate-intensity physical activity per day (in minutes); MVPA, moderate-to-vigorous-intensity physical activity; SB, sedentary behavior; SST, sedentary screen time; TPA, total physical activity; VPA, vigorous-intensity physical activity per day (in minutes); WAZ, weight-for-age z-score. Data are presented as mean (95% confidence interval). Bold values indicate statistical significance at P < 0.05.

*Differences were tested using Kruskal-Wallis tests by residential setting. Analytical sample: a n = 112*, Urban *(n = 74): BMI < normal = 2, BMI = normal = 38, BMI > normal = 34; Rural (n = 38): BMI < normal = 3, BMI = normal = 31, BMI > normal = 4.*

€: P-value between BMI < normal and BMI = normal; π: P-value between BMI < normal and BMI > normal; β: P-value between BMI = normal and BMI > normal. **ϵ²** : epsilon squared

### 3.1 Anthropometric characteristics

In both urban and rural subsamples, significant differences were observed across BMI categories for several anthropometric indicators, particularly weight, HAZ and BAZ.

In the urban group, children classified as BMI > normal exhibited significantly higher body weight, BMI, HAZ, and BAZ compared with children in the BMI < normal and BMI = normal categories. Pairwise comparisons confirmed significant differences between:

BMI < normal and BMI > normal for weight, BMI, HAZ, and BAZ,
BMI = normal and BMI > normal for BMI and BAZ.

These findings reflect expected variations in growth status across BMI classifications. However, age and height did not significantly differ between BMI groups in the urban setting.

Similarly, in the rural group, significant differences were observed for weight, BMI, HAZ, and BAZ across BMI categories. Children in the BMI > normal category had higher anthropometric indices compared with their normal-weight and underweight peers. Pairwise comparisons indicated significant differences particularly between BMI < normal and BMI > normal, and between BMI = normal and BMI > normal for BMI and BAZ (**Table 1**).

No statistically significant differences were observed for weight-for-age z-score (WAZ) in either residential setting.

Given the observational design and the very small number of children in the BMI < normal category (urban n = 2; rural n = 3), these anthropometric differences should be interpreted cautiously. The limited size of some subgroups may reduce statistical power and affect the stability of pairwise comparisons.

### 3.2 Movement behaviours

Regarding 24-hour MB (light-intensity physical activity (LPA), moderate-intensity physical activity (MPA), vigorous-intensity physical activity (VPA), moderate-to-vigorous physical activity (MVPA), total physical activity (TPA), sedentary behaviour (SB) (screen sedentary time, and sleep duration), no statistically significant differences were observed across BMI categories within either urban or rural settings.

In the urban subsample, movement behaviour variables did not significantly differ between BMI groups, although SB approached statistical significance (p = 0.053), with children in the normal BMI category showing slightly higher sedentary time compared with those in the BMI > normal category. However, this difference did not reach conventional statistical significance and should not be overinterpreted.

In the rural subsample, children with BMI > normal tended to show higher mean values for MVPA and TPA and lower sedentary time compared with other BMI groups; however, these differences were not statistically significant. Similarly, screen sedentary time and sleep duration did not differ significantly across BMI categories in either setting.

Overall, the absence of statistically significant differences in MB suggests that BMI classification was not strongly associated with variations in PA, SB, or sleep within this sample. However, the small size of certain subgroups particularly BMI < normal and BMI > normal in rural areas may have limited the ability to detect meaningful associations. Given the observational and cross-sectional design of the study and the limited size of some BMI subgroups, these findings should therefore be interpreted with caution.

**Table 2** presents the number and proportion of preschool children meeting the 24-hour movement guidelines, stratified by BMI categories and residential setting. In the total sample (n = 89 for accelerometer-derived variables), 76.4% of children achieved the recommendation of at least 60 minutes per day of MVPA. In contrast, only 40.5% met the recommendation of at least 80 minutes per day of TPA. Regarding SB and sleep (n = 112), 52.7% of children met the screen time guideline (≤ 60 minutes per day), whereas 81.3% achieved the recommended sleep duration of 10–13 hours per day. Overall, only 18.0% of children met all five integrated 24-hour movement recommendations.

**Table 2:** Number and proportion of preschool children meeting 24-hour movement guidelines stratified by BMI Categories by Region.

		Urban (51) (57.30 %)				*Rural (38)* (42.70 %)			
Variables	Total n (%)	BMI < normal (n=1) (1.96 %)	BMI = normal (n=29) (56.86 %)	*BMI > normal* *(n=21)* (41.18 %)	*P*	BMI < normal (n=3) (7.90 %)	BMI = normal (n=31) (81.58%)	*BMI > normal* *(n=4)* (10.53%)	*P*
≥ 60 min/day of MVPA, n = 89	68 (76.4%)	–	20 (68.96 %)	16 (76.19 %)	**<0.001**	3 (100 %)	25 (80.64 %)	4 (100 %)	0.092
≥ 80 min/day of TPA, n = 89	36 (40.5%)	–	6 (20.69 %)	8 (38.10 %)	**<0.001**	1 (33.33 %)	17 (54.84%)	4 (100 %)	**0.011** 0.591€ **0.036Π** **0.006β**
≥ 60 min/day of MVPA and ≥ 180 min/day of TPA, n = 89	36 (40.5%)	–	6 (20.69 %)	8 (38.10 %)	**<0.001**	1 (33.33 %)	17 (54.84%)	4 (100 %)	**0.011** 0.591€ **0.036Π** **0.006β**
≤ 60 min/day of SST, n a = 112	59 (52.7%)	1 (50 %)	21 (55.26 %)	16 (47.06 %)	0.487	1 (33.33 %)	15 (48.39%)	4 (100 %)	**0.009** 1.000€ **0.036Π** **0.003β**
10–13 h/day of sleep, n a= 112	91 (81.3%)	2 (100 %)	31 (81.58 %)	27 (79.41 %)	0.817	3 (100 %)	24 (77.42 %)	4 (100 %)	0.092
Meeting all 5 recommendations, n = 89	16 (18.0%)	–	2 (6.90 %)	3 (14.28 %)	**<0.001**	0 (0 %)	7 (22.58 %)	4 (100 %)	**<0.001** 1.000€ **0.004Π** **<0.001β**

MVPA, moderate-to-vigorous-intensity physical activity; SST, sedentary screen time; TPA, total physical activity. Analytical sample *a n = 112*, Urban *(n = 74): BMI < normal = 2, BMI = normal = 38, BMI > normal = 43; Rural (n = 38): BMI < normal = 3, BMI = normal = 31, BMI > normal = 4.* €: P-value between BMI < normal and BMI = normal ; π: P-value between BMI < normal and BMI > normal ; β: P-value between BMI = normal and BMI > normal. Meaningful effects (criteria: p < 0.05) are highlighted in bold.

In the urban subsample, a statistically significant association between BMI categories and compliance with certain recommendations was observed. Significant differences were found for achieving at least 60 minutes per day of MVPA (P < 0.001), at least 80 minutes per day of TPA (P < 0.001), the combined MVPA and TPA recommendation (P < 0.001), and meeting all five recommendations simultaneously (P < 0.001). These proportions suggest higher compliance with PA recommendations among children with BMI > normal compared with those with normal BMI. However, no significant differences were observed across BMI categories for screen time (P = 0.487) or sleep duration (P = 0.817).

In the rural subsample, significant differences across BMI categories were also observed for achieving at least 80 minutes per day of TPA (P = 0.011), for the combined MVPA and TPA recommendation (P = 0.011), for meeting the screen time guideline (P = 0.009), and for adherence to all five recommendations (P < 0.001). In contrast, no significant differences were found for MVPA alone (P = 0.092) or sleep duration (P = 0.092). The observed proportions, particularly in the BMI > normal category, should be interpreted cautiously due to the small size of this subgroup (n = 4).

Given the observational and cross-sectional design of the study, these findings should be interpreted with caution. The small size of certain subgroups, particularly the BMI < normal and BMI > normal categories in the rural setting, may have limited statistical power and the stability of the proportional estimates. Therefore, the observed associations require confirmation in larger samples.

**Table 3** presents executive function and motor skill outcomes of preschool children stratified by BMI categories within urban and rural settings. Overall, no significant differences across BMI categories were observed for EF or motor skill outcomes in either residential setting. Indeed, in the urban subsample, no significant differences across BMI categories were observed for inhibition (P = 0.817) or working memory (P = 0.998). Similarly, no significant differences across BMI categories were observed for functional mobility (P = 0.565), postural steadiness (P = 0.219), lower body strength (P = 0.486), upper body strength (P = 0.208), or dexterity (P = 0.615). Furthermore, in the rural subsample, no significant differences across BMI categories were observed for inhibition (P = 0.547) or working memory (P = 0.432). Likewise, no significant differences across BMI categories were observed for functional mobility (P = 0.943), postural steadiness (P = 0.149), lower body strength (P = 0.448), upper body strength (P = 0.304), or dexterity (P = 0.116).

**Table 3:** Executive function and gross and fine motor skill outcomes of preschool children stratified by BMI Categories by Region (Mean ± SD).

		Urban (74)					Rural (38)				
Variables	Total (n = 112)	BMI < normal (n=2) [95% CI]	BMI = normal (n=38) [95% CI]	BMI > normal (n= 34) [95% CI]	*P*	ϵ²	BMI < normal (n= 3) [95% CI]	BMI = normal (n= 31) [95% CI]	BMI > normal (n= 4) [95% CI]	*P*	ϵ²
Inhibition	0.67 ± 0.24	0,52 ± 0,30 [0.63; 0.71]	0,63 ± 0,24 [-2.18; 3.22]	0,64 ± 0,25 [0.55; 0.71]	0.817	-0.022	0,85 ± 0,18 [0.40; 1.30]	0,73 ± 0,24 [0.64; 0.82]	0,68 ± 0,24 [0.30; 1.06]	0.547	-0.023
Working memory	1.89 ± 0.74	1,83 ± 1,18 [1.75; 2.03]	1,78 ± 0,74 [-8.77; 12.43]	1,78 ± 0,69 [1.54; 2.02]	0.998	-0.028	1,78 ± 0,38 [0.84; 2.72]	2,16 ± 0,74 [1.89; 2.43]	1,83 ± 0,88 [0.43; 3.23]	0.432	-0.09
Functional mobility (s)	4.88 ± 1.33	5,50 ± 0,71 [4.63; 5.13]	4,82 ± 1,50 [-0.88; 11.88]	4,94 ± 1,50 [4.33; 5.31]	0.565	-0.012	4,67 ± 0,58 [3.23; 6.11]	4,90 ± 1,01 [4.53; 5.27]	4,75 ± 1,26 [2.75; 6.75]	0.943	-0.054
Postural steadiness (s)	15.59 ± 9.11	5,00 ± 4,24 [13.88; 17.30]	13,68 ± 8,23 [-33.09; 43.09]	14,59 ± 10,01 [10.97; 16.39]	0.219	0.015	17,67 ± 11,72 [-11.44; 46.78]	20,03 ± 7,83 [17.16; 22.90]	11,50 ± 7,85 [-0.99; 23.99]	0.149	-0.052
Lower body strength (cm)	58.24 ± 20.80	37,00 ± 8,49 [54.35; 62.13]	51,92 ± 20,33 [-39.28; 113.28]	51,32 ± 19,56 [45.24; 58.60]	0.486	-0.008	85,67 ± 24,83 [23.99; 147.35]	70,35 ± 14,79 [64.92; 75.78]	73,25 ± 12,69 [53.06; 93.44]	0.448	-0.011
Upper body strength (kg)	8.28 ± 2.73	4,75 ± 3,18 [7.77; 8.79]	7,92 ± 2,61 [-23.82; 33.32]	7,54 ± 2,91 [7.06; 8.78]	0.208	0.016	8,17 ± 2,08 [3.00; 13.34]	9,76 ± 2,12 [8.98; 10.54]	8,25 ± 2,84 [3.73; 12.77]	0.304	0.011
Dexterity (s)	37.32 ± 9.28	44,50 ± 6,36 [35.58; 39.06]	38,92 ± 9,48 [-12.64; 101.64]	39,50 ± 10,06 [35.80; 42.04]	0.615	-0.014	31,00 ± 2,65 [24.42; 37.58]	33,13 ± 7,69 [30.31; 35.95]	37,25 ± 3,50 [31.68; 42.82]	0.116	0.066

€: P-value between BMI < normal and BMI = normal; π: P-value between BMI < normal and BMI > normal ; β: P-value between BMI = normal and BMI > normal ; **ϵ²** : epsilon squared.

Differences were tested using Kruskal-Wallis tests.

Given the observational and cross-sectional design of the study, these findings should be interpreted with caution. In addition, the very small number of children in the BMI < normal category and the limited size of the BMI > normal subgroup, particularly in the rural setting, may have reduced statistical power and limited the ability to detect small associations. Therefore, the absence of statistically significant differences does not necessarily indicate the absence of meaningful relationships, and these results warrant confirmation in larger samples.

## 4 Discussion

The present cross-sectional observational study aimed to examine urban–rural differences in BMI categories, 24-hour movement guideline compliance, EF, and MS among Tunisian preschool children aged 4 to 5 years. To our knowledge, this is the first study in Tunisia to concurrently investigate these four interrelated domains within an integrated analytical framework.

Overall, significant differences were observed between BMI categories for anthropometric indicators in both urban and rural settings. However, BMI was not significantly associated with EF or motor skill outcomes in either residential context. In contrast, significant associations emerged between BMI categories and adherence to certain 24-hour movement recommendations, particularly in the urban subsample and partially in the rural group. These findings suggest that weight status in early childhood was observed to be associated with behavioural compliance patterns rather than with motor or cognitive performance at this age.

Given the observational and cross-sectional design, findings should be interpreted as associational rather than causal.

### 4.1 Anthropometric differences according to BMI categories

Statistically significant differences were observed between BMI categories for most anthropometric variables in both urban and rural settings. Children classified as having a BMI above normal exhibited significantly higher body weight, BMI, and BAZ compared with peers of normal or below-normal weight. Significant differences were also found for HAZ, particularly in comparisons between normal BMI and BMI above normal, suggesting a more pronounced linear growth in children with elevated BMI.

These differences are partly inherent to BMI-based classification and should be interpreted with caution, given the recognized limitations of BMI in early childhood. Indeed, BMI does not differentiate between fat mass and lean mass and reflects overall somatic growth more than isolated adiposity. Similar observations have been reported (Ltifi et al., 2026), where children with BMI above normal also showed higher linear growth indicators, suggesting that BMI captures a global body development profile rather than strict excess adiposity (Group, 2006a; Bornstein et al., 2012; Krupsky et al., 2020).

In our sample, age did not differ significantly across BMI categories in urban (p = 0.912) or rural (p = 0.080) settings, although the latter value approached the significance threshold. The absence of substantial age differences indicates that observed anthropometric variations are primarily associated with weight status rather than maturational differences (Nervik et al., 2011; Okely et al., 2021; Rico-González et al., 2024). This aligns with previous findings showing that, in preschool-aged children, differences in height and weight across BMI categories largely reflect individual growth trajectories rather than chronological age effects (Tandon et al., 2019; Whiting et al., 2021; Rico-González et al., 2024).

### 4.2 BMI and 24-hour movement behaviours

Analysis of continuous accelerometery-derived variables (LPA, MPA, VPA, MVPA, TPA, SB, and sleep) revealed no statistically significant differences between BMI categories in either urban or rural contexts. In the urban setting, a trend toward lower sedentary time in children with BMI above normal was observed, though it did not reach statistical significance. In the rural setting, children with BMI above normal exhibited higher mean MVPA and TPA, but these differences remained non-significant.

These results suggest that, at 4–5 years of age, daily volumes of PA and SB do not yet differ substantially according to weight status. This observation is consistent with literature indicating that associations between BMI and PA strengthen with age, particularly from primary school onward, when behavioural autonomy increases and lifestyle differences accumulate (Chaput et al., 2014; Pate et al., 2015; Kuzik and Carson, 2016; Tremblay et al., 2016).

In the Tunisian context, preschool environments are generally characterized by structured routines, guided activities, and relatively homogeneous schedules. Such environmental constraints may attenuate inter-individual differences related to body composition (Timmons et al., 2007; Ltifi et al., 2025c). MB in early childhood appear to be influenced more by educational and family environments than by weight status perse (de Souza Morais et al., 2021; Whiting et al., 2021).

Furthermore, although 76.4% of children met the recommendation of ≥ 60 min/day of MVPA and 81.3% met the recommended sleep duration (10–13 h/day), only 18% adhered to all five integrated 24-hour movement guidelines. This low rate of combined adherence aligns with international data notably from the SUNRISE project (Delisle Nyström et al., 2020).

The substantial discrepancy between high compliance with MVPA recommendations (76.4%) and the very low proportion of children meeting all integrated 24-hour movement guidelines (18%) suggests that other behavioural components, particularly sedentary screen time and light-intensity physical activity, are the main limiting factors for full guideline adherence. In other words, while children appear sufficiently active in moderate-to-vigorous intensity domains, excessive sedentary behaviour and/or insufficient light activity may explain the low overall compliance with the combined guidelines. This pattern highlights the importance of considering movement behaviours as an integrated 24-hour system rather than isolated components.

This finding is consistent with recent person-centered studies conducted in comparable contexts within the Middle East and Gulf region, which have shown that children’s health behaviours tend to cluster, with screen time, sleep quality, and dietary habits being stronger determinants of overall behavioural profiles than PA alone. For example, studies in school-aged populations in the United Arab Emirates have identified distinct health behaviour profiles where screen time and sleep are key predictors of health outcomes and school satisfaction (Webster et al., 2024; Webster et al., 2025; Meksi et al., 2026). Although these studies were conducted in older children, they support the idea that sedentary behaviours and lifestyle patterns may play a central role in shaping overall health behaviour profiles in youth.

It is important to note that the study may have been underpowered to detect small-to-moderate associations due to the limited number of participants in specific BMI subgroups, particularly within the rural BMI < normal and BMI > normal categories. Therefore, non-significant findings should be interpreted cautiously, as they may reflect limited statistical sensitivity rather than true absence of associations.

### 4.3 Adherence to 24-hour guidelines and urban–rural differences

Unlike continuous analyses, categorical analyses of guideline adherence revealed statistically significant associations between BMI categories and compliance with specific recommendations, particularly in the urban subsample. Significant differences were observed for achieving at least 60 minutes per day of MVPA, at least 80 minutes per day of TPA, the combined MVPA + TPA recommendation, and adherence to all five integrated recommendations. The proportions indicate higher compliance among children with BMI above normal compared with their normal-weight peers. No significant differences were observed for screen time or sleep duration.

These findings suggest that weight status may be more closely associated with threshold-based compliance rather than absolute activity volumes, as dichotomous analyses revealed differences not captured in continuous measures (Pate et al., 2006; Pate et al., 2015; Okely et al., 2021). As highlighted in recent literature, the use of binary indicators (compliant vs non-compliant) can reduce analytical sensitivity and obscure gradual behavioural variations (Pate et al., 2006; Pate et al., 2015).

In the urban environment, structured routines, access to clubs, and sports infrastructure may facilitate adherence, particularly among children with higher BMI, suggesting that environmental context can modulate behavioural patterns. In contrast, rural children showed significant differences for TPA, combined MVPA + TPA, screen time, and overall guideline adherence, but no significant differences were observed for MVPA alone or sleep. The small size of the BMI > normal subgroup (n = 4) limits the stability of proportional estimates and increases the risk of type I or II errors.

In the total sample, 76.4% of children achieved the recommendation of ≥ 60 minutes per day of MVPA, whereas only 40.5% met the recommendation of ≥ 80 minutes per day of TPA, a proportion identical to that observed for the combined MVPA + TPA recommendation. Regarding SB and sleep, 52.7% of children met the screen time guideline (≤ 60 min/day), and 81.3% met the recommended sleep duration (10–13 h/day). Overall, only 18% of children adhered to all five integrated 24-hour movement guidelines. These results indicate that, although adherence to individual recommendations may be relatively high, compliance with the full set of guidelines remains limited, highlighting the need for integrated interventions (Group, 2006b; WHO Guidelines Approved by the Guidelines Review Committee, 2019; Okely et al., 2021; Ltifi et al., 2025d).

Overall, these findings underscore the importance of residential context, Urban environments may be associated with greater opportunities for structured physical activities, whereas rural settings may be associated with more opportunities for spontaneous outdoor play (Ltifi et al., 2025a; Ltifi et al., 2025c). Future research should incorporate detailed environmental variables and larger sample sizes to better understand these patterns and their implications for early childhood health (Ltifi et al., 2026).

### 4.4 BMI, executive functions, and motor competence

No statistically significant differences were observed between BMI categories for EF (inhibition and working memory) or for gross and fine motor components in either urban or rural contexts. These findings contrast with studies in older children showing lower motor or cognitive performance in overweight or obese children (Barros et al., 2021; Fernandes et al., 2022) but align with other reports indicating no clear associations in preschool-aged children (Fernandes et al., 2022; Ltifi et al., 2026).

Several mechanisms can be considered. Physiologically, elevated BMI could theoretically influence motor performance via musculoskeletal or postural constraints. Neurocognitively, metabolic or nutritional differences could affect early brain development (National Research C and Institute of Medicine Committee on Integrating the Science of Early Childhood D, 2000; Brown and Jernigan, 2012). Behaviourally, variations in PA, sleep, or sedentary patterns could modulate developmental trajectories (Tremblay et al., 2011; Chaput et al., 2014; Tremblay et al., 2017). However, in our sample, these potential mechanisms did not translate into measurable differences, suggesting that, at this age, environmental factors (structured routines, educational supervision) may play a protective or buffering role.

Furthermore, the very small subgroup sizes BMI < normal (urban n = 2; rural n = 3) and BMI > normal (n = 4) strongly limit statistical power and the ability to detect small associations. This limitation should be carefully considered when interpreting the results, and future studies with larger samples are needed to confirm these findings.

### 4.5 Public health implications in Tunisia

Even in the absence of significant associations between BMI, MB, EF, and motor competence, the low proportion of children meeting all 24-hour recommendations (18%) represents an important public health signal. These results suggest that interventions in Tunisia should adopt a universal approach rather than targeting only weight status (WHO Guidelines Approved by the Guidelines Review Committee, 2019; Bull et al., 2020; Okely et al., 2021).

Promoting preschool environments that facilitate both structured and unstructured PA, raising parental awareness regarding screen time and sleep hygiene, and considering urban–rural specificities in program design appear pertinent (Ltifi et al., 2025b; Ltifi et al., 2025c). Early childhood represents a critical developmental window during which establishing balanced lifestyle habits can have lasting effects on weight trajectory and neuro-motor development (Diamond, 2013; Whiting et al., 2021; Reisberg et al., 2024).

### 4.6 Study limitations

This study has several limitations that should be considered when interpreting results. First, the cross-sectional observational design does not allow causal inferences between BMI, MB, EF, and motor competence. The convenience sample, without *a priori* power calculation, may limit generalizability and increase the risk of type II errors. In addition, no *a priori* power calculation was performed, which limits the ability to determine whether the study was sufficiently powered to detect small-to-moderate effects. Very small subgroup sizes, notably BMI < normal and BMI > normal in rural settings, reduce estimate stability and may have hindered detection of small associations. The presence of very small cell sizes in specific BMI × residential subgroups (e.g., BMI < normal and BMI > normal in rural settings) further reduces statistical power and may increase the likelihood of type II error. Consequently, the predominance of non-significant associations should not be interpreted as evidence of absence of relationships but rather as inconclusive findings requiring confirmation in larger, adequately powered samples. Aggregation of “at risk of overweight,” “overweight,” and “obese” categories into a single BMI > normal group, although methodologically justified due to subgroup sizes, may mask internal heterogeneity. Analyses were not adjusted for potential confounders such as socioeconomic status, parental education, dietary intake, or accelerometer wear time. Executive function assessment was limited to behavioural inhibition and visuospatial working memory. Although the Arabic version of the Early Years Toolbox was well understood by all participants, no comprehension or administration difficulties were observed during testing, and each assessment included a standardized practice phase before formal evaluation. Nevertheless, the Arabic version is still undergoing formal psychometric validation in Tunisian preschool children. Consequently, reduced measurement sensitivity cannot be excluded, and the absence of significant associations involving executive function should be interpreted cautiously, as it may partly reflect measurement limitations rather than a true absence of relationships. In addition, although familiarization sessions were conducted and no behavioral issues were reported, the possibility that awareness of being monitored may have minimally influenced children’s habitual PA cannot be completely excluded.

Moreover, although analyses were stratified by urban and rural residence, the study was not sufficiently powered to formally test interaction effects between BMI category and residential setting. Consequently, it cannot be concluded whether residential context truly moderates the observed associations. These analyses should therefore be considered exploratory and hypothesis-generating, requiring confirmation in larger studies specifically designed to evaluate interaction effects.

Finally, while accelerometery provides objective measurement, it does not capture contextual activity quality. Longitudinal and interventional studies integrating mediators (actual activity intensity, sleep quality, dietary habits) and moderators (family context, preschool environment) are needed to better understand mechanisms underlying the relationships between BMI, MB, and neuro-motor development.

## 5 Conclusions

This cross-sectional study is the first to examine urban–rural differences in BMI categories, 24-hour movement guideline compliance, executive functions, and motor skills among Tunisian preschool children. Significant differences were observed across BMI categories for anthropometric measures, but no significant associations were identified between BMI categories and motor or executive function outcomes. However, these findings should be interpreted cautiously, as the absence of statistically significant associations should not be considered evidence of the absence of relationships, particularly given the limited size of some subgroups and the available statistical power. In contrast, differences in compliance with certain 24-hour movement recommendations were observed across BMI categories, particularly in urban settings. These findings suggest potential associations between weight status and specific movement-related behaviours rather than demonstrated effects on neuro-motor performance. However, the direction and mechanisms underlying these associations remain unclear. Given the observational and cross-sectional design of this study, causal relationships between BMI, movement behaviours, executive functions, and motor competence cannot be established. The low proportion of children meeting all integrated 24-hour movement recommendations (18%) highlights the importance of promoting healthy movement behaviours from early childhood, including sufficient physical activity, adequate sleep duration, and appropriate screen-time management, through context-specific strategies. Future longitudinal and intervention studies involving larger and more representative samples, as well as adjustment for potential confounding factors (e.g., family context, socioeconomic status, and dietary behaviours), are needed to better understand the mechanisms underlying the relationships between BMI, movement behaviours, and cognitive-motor development during early childhood.

## Data Availability

The datasets are confidential to protect participants’ privacy and are not publicly available. However, they can be obtained from the first author, Mohamed Amine Ltifi (mohamedltifi19@gmail.com; medamine.ltifi@issepgf.ugaf.tn), upon reasonable request and in accordance with applicable ethical requirements.
